# Phylogenetic relationship based on DNA barcodes and comparative analysis of phytochemical contents among *Rhynchostylis* orchids in Thailand

**DOI:** 10.1038/s41598-026-44785-x

**Published:** 2026-03-18

**Authors:** Juthaporn Saengprajak, Jirapa Phetsom, Aphidech Sangdee, Arnusorn Saengprajak, Thanwanit Thanyasiriwat, Wuttipong Mahakham

**Affiliations:** 1 Department of Biology, Faculty of Science, Mahasarakham University, Kantarawichai District, Mahasarakham, 44150 Thailand; 2 Department of Physics, Faculty of Science, Mahasarakham University, Kantarawichai District, Mahasarakham, 44150 Thailand; 3 Plant Genome and Disease Research Unit, Department of Agriculture and Resources, Faculty of Natural Resources and Agro-Industry, Kasetsart University Chalermphrakiat Sakon Nakhon Province Campus, Sakon Nakhon, 47000 Thailand; 4 Department of Biology, Faculty of Science, Khon Kaen University, Khon Kaen, 40002 Thailand

**Keywords:** Antioxidant activity, ITS region, *matK* gene, Orchidaceae, Phylogeny, Phytochemical profiling, *Rhynchostylis*, Ecology, Ecology, Plant sciences

## Abstract

*Rhynchostylis* orchids are native to Thailand and have considerable commercial importance. However, species delimitation remains difficult because of overlapping morphology, environmental plasticity, short flowering periods, and extensive horticultural hybridization. In addition, comprehensive molecular and phytochemical data for the genus remain limited. This study examined phylogenetic relationships among nine *Rhynchostylis* accessions and one *Aerides* species using the internal transcribed spacer (ITS) and *maturase K* (*matK*) DNA-barcode regions, and further evaluated their phytochemical properties to support species identification, conservation, and superior genotype selection. The ITS and *matK* regions were amplified and sequenced, and phylogenetic trees were reconstructed using maximum likelihood (ML) and Neighbour-joining (NJ) approaches. These analyses generally recovered *R. retusa* and *R. coelestis* as distinct lineages from the *R. gigantea* complex. ITS showed slightly higher discriminatory power among closely related *R. gigantea* forms, whereas *matK* provided complementary phylogenetic structure. When interpreted together with diagnostic floral traits, these molecular data strengthened species delimitation within the genus. Phytochemical profiling of ethanolic leaf extracts revealed significant interspecific variation, with total phenolic content (TPC), total flavonoid content (TFC), and 2,2′-diphenyl-1-picrylhydrazyl (DPPH) radical-scavenging activity ranging from 2.30 ± 0.32 to 11.38 ± 0.01 mg GAE/g DW, 1.47 ± 0.62 to 18.14 ± 0.33 mg RE/g DW, and 12.91 ± 0.09 to 42.17 ± 0.33 mg TE/g DW, respectively. *R. gigantea* ‘Chang Phlai’ consistently exhibited the highest phenolic content and antioxidant capacity, highlighting its potential pharmacological relevance. Collectively, this study demonstrates that integrating DNA barcoding with floral morphology and phytochemical evidence provides a baseline molecular and chemical framework that helps clarify taxonomic interpretation in *Rhynchostylis*. It may also inform future conservation assessments and sustainable utilization efforts for these economically and medicinally important orchids.

**Supplementary Information:**

The online version contains supplementary material available at 10.1038/s41598-026-44785-x.

## Introduction

*Rhynchostylis* orchids, members of the Orchidaceae family, are tropical epiphytes known for their waxy flowers and distinctive fragrance. The genus is distributed across South and Southeast Asia, including India, Nepal, Myanmar, Thailand, Laos, Vietnam, and southern China, where these plants typically grow on trees in humid forest environments and are characterized by their long, dark green leaves^1^. Thailand hosts approximately 1,300 orchid species across nearly 190 genera and supports a major ornamental orchid industry, with about 54% of cultivated orchids exported. Within this sector, *Rhynchostylis* is regarded as an economically important genus^2^.

The genus *Rhynchostylis*, commonly referred to as the foxtail orchid, is taxonomically distinguished by its characteristic column structure, a feature reflected in its name, derived from the Greek words “rhyncho” (beak) and “stylis” (column). *Rhynchostylis* Blume is a small orchid genus comprising five accepted species according to the Plants of the World Online database (Royal Botanic Gardens, Kew): *Rhynchostylis coelestis* (Rchb.f.) A.H.Kent, *R. cymifera* Yohannan, J.Mathew & Szlach., *R. gigantea* (Lindl.) Ridl., *R. retusa* (L.) Blume, and *R. rieferi* W.E.Higgins^3^. Among these, *R. gigantea* is further subdivided into two subspecies^4^.

In Thailand, *R. gigantea* and *R. retusa* are the most prevalent, with *R. gigantea* particularly valued for its diverse flower coloration and popularity in cultivation and hybridization^5^. However, species identification within *Rhynchostylis* remains challenging due to morphological similarities, coupled with variations influenced by environmental conditions and developmental stages^6,7^. Misidentification often occurs due to the highly similar external characteristics among species, as well as distinctions between juvenile and mature forms. This difficulty is further exacerbated by the brief flowering period of *Rhynchostylis*, which typically flowers only between January and February, as well as by seasonal limitations and increasing influence from climatic variation^8,9^. Such uncertainty is particularly significant given the differing economic and medicinal importance attributed to different species^10^. Furthermore, the decline in genetic diversity has raised conservation concerns, leading to the inclusion of *Rhynchostylis* in Appendix II of CITES^11^.

The genus *Aerides* is phylogenetically close to *Rhynchostylis* within the subtribe Aeridinae and forms a distinct evolutionary lineage. The two genera share several morphological features that may cause taxonomic ambiguity when classifications rely solely on morphology^12,13^. Previous studies have consistently placed *Aerides* and *Rhynchostylis* in adjacent lineages within Aeridinae^12,14,15^, although they are treated as separate genera based on morphological and molecular evidence.

However, the taxonomic delimitation of *Rhynchostylis* remains problematic^16^. The species share highly similar vegetative morphology and are often distinguishable only during the flowering stage. Because the blooming period is brief and increasingly influenced by climatic variation, reliable identification based solely on leaf and stem characters is difficult. In addition, many morphological traits exhibit strong environmental plasticity, such that individuals of the same species may appear markedly different under different growing conditions. *Rhynchostylis* also flowers only once or twice a year for a short period, further limiting access to diagnostic reproductive characters^17^. Moreover, widespread horticultural hybridization within *R. gigantea* has produced numerous intermediate forms that obscure natural species boundaries^18^. Taken together, these factors contribute to frequent misidentification and persistent uncertainty in species delimitation within the genus.

*Rhynchostylis* species share a characteristic vegetative syndrome: all are epiphytic and monopodial, with short stems, thick aerial roots, and distichous, strap-shaped, coriaceous leaves with bilobed apices^19^. These traits are useful at the generic level and distinguish *Rhynchostylis* from related vandaceous genera such as *Vanda* and *Aerides*^19,20^. Species delimitation, however, relies mainly on floral characters, particularly labellum structure and inflorescence architecture^21,22^. Other traits, including flower color and size, are often variable and influenced by environmental conditions or hybridization^7,9,22^. Given this variability and the short flowering period of the genus, molecular approaches such as DNA barcoding provide important complementary evidence for accurate identification. Although molecular approaches have been applied to *Rhynchostylis* and related *Aerides*-*Vanda* orchids (12, 13, 19), most studies have examined only a limited range of taxa or relied on a single line of evidence, rather than adopting a fully integrative framework.

DNA barcoding provides a useful complementary approach in this system because sequence variation in nuclear and plastid loci is genetically stable, heritable, and largely independent of environmental influences^18^. Comparative analysis of the ITS and *matK* regions can therefore delineate genetic boundaries even where morphology is ambiguous or overlapping^23^. This is particularly relevant in *Rhynchostylis*, where species delimitation, hybrid detection, and recognition of closely related taxa remain difficult when based solely on morphology. DNA barcoding has consequently become an important tool in plant taxonomy, biodiversity assessment, and genetic resource conservation^24,25^.

In plants, barcoding typically relies on nuclear and chloroplast regions such as ITS, *matK*, *rbcL*, *atpF-atpH*, *psbK-psbI*, and *trnH-psbA*, rather than the mitochondrial COI gene widely used in animals^26,27^. Among these, *rbcL* is conserved and easily amplified but offers limited resolution, whereas ITS and *matK* exhibit higher sequence variability and discriminatory power^26–28^. Empirical studies support this pattern: Kim et al. (2014) successfully identified 89 orchid species using DNA barcodes^29^, and Parveen et al. (2017) showed that ITS and *matK* provided strong discrimination in *Rhynchostylis retusa* and other Indian orchids^27^. In contrast, Chattopadhyay et al. (2017) found *matK* and *rbcL* insufficient for resolving closely related *Dendrobium* species, whereas ITS achieved higher species-level resolution^29^. Similarly, Raskoti & Ale (2021) identified ITS as the most effective barcode for medicinal orchids in Asia, with the ITS + *matK* combination performing best^25^. Additional studies in *Paphiopedilum* and *Dendrobium* have also demonstrated the usefulness of multilocus barcoding approaches^30,31^. On this basis, ITS and *matK* were selected for the present study, as previous work indicates that these loci provide stronger species discrimination than *rbcL*, particularly in orchids^24,27^.

In parallel with the genetic analyses, profiling phenolic composition provides important insight into the pharmacological potential of plant extracts. Phenolic compounds, particularly flavonoids and phenolic acids, are widely recognized for their antioxidant, anti-inflammatory, and anticancer activities^32^. In this study, ethanolic leaf extracts of *Rhynchostylis* and related taxa were evaluated for total phenolic content (TPC), total flavonoid content (TFC), and antioxidant capacity using the 2,2′-diphenyl-1-picrylhydrazyl (DPPH) radical-scavenging assay. Previous work likewise supports the bioactive potential of these orchids. Noorjahan et al. (2024) identified diverse medicinally relevant compounds in root extracts of *R. retusa* from the Eastern Ghats of India^33^, and Kumari et al. (2024) reported high polyphenolic levels and therapeutic promise in the same species^32^. Comparable antioxidant properties have also been documented in wild orchids from Nepal^34^, polysaccharides of *Dendrobium* species in Manipur^35^, and methanolic extracts of *Phalaenopsis* tissues^36^.

This study aims to examine species boundaries and evolutionary relationships within *Rhynchostylis* in Thailand using an integrative approach. Molecular phylogenetic analyses based on ITS and *matK* were used to resolve genetic relationships, while phytochemical profiling evaluated whether variation in phenolic composition and antioxidant activity corresponds to genetic differentiation among taxa. By integrating these datasets, the study provides a clearer basis for taxonomic delimitation, explores links between evolutionary divergence and biochemical traits, and contributes information relevant to conservation and pharmacological evaluation of *Rhynchostylis*.

## Materials and methods

### Plant materials

**Fig. 1:**
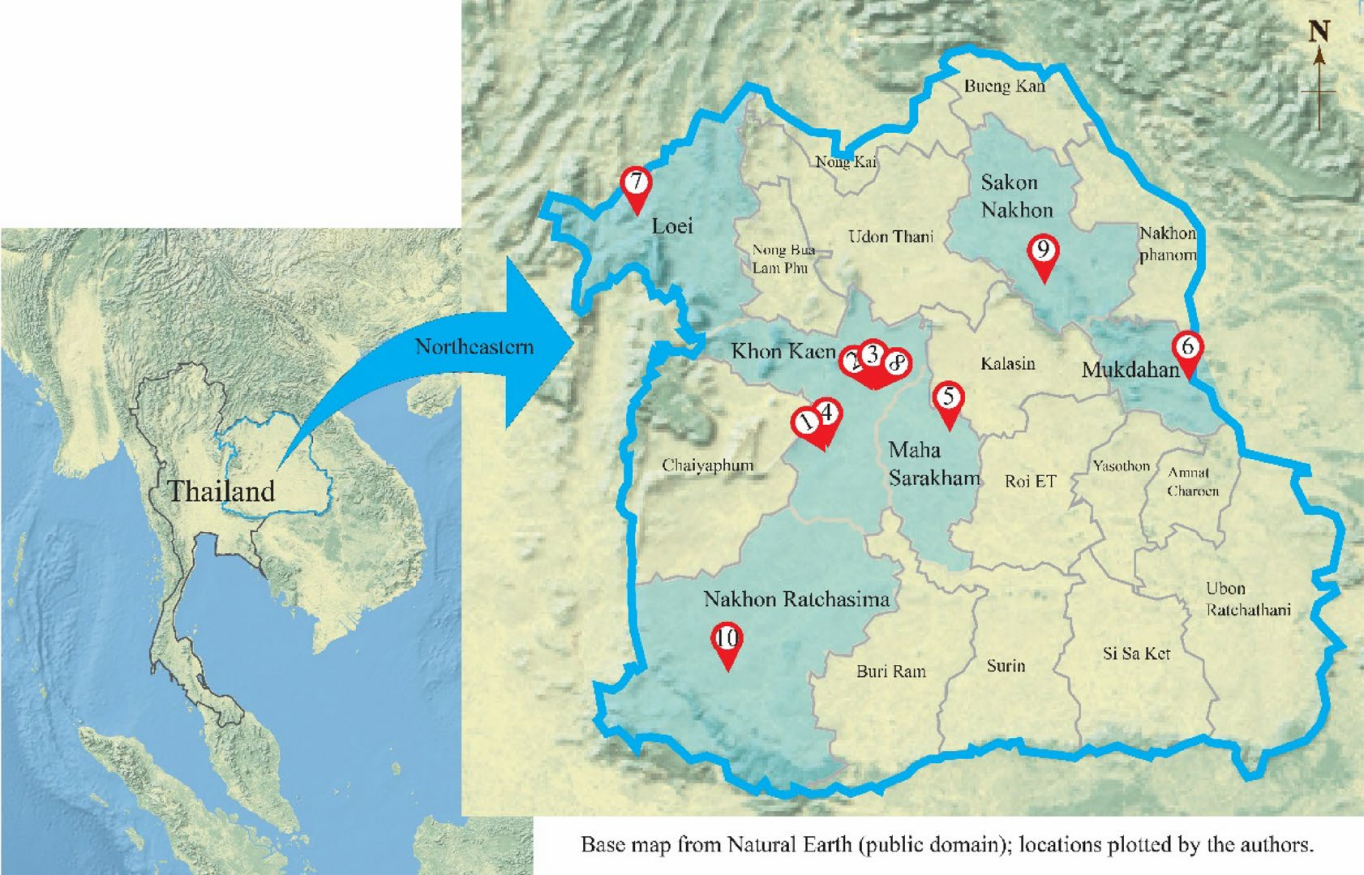
Sample collection sites in northeastern Thailand. Locations are indicated as follows: 1, Wat Pa Mancha Khiri, Mancha Khiri District, Khoneun Kaen Province; 2, 3, and 8, Khon Kaen University, Mueang District, Khon Kaen Province; 4, Mancha Khiri District, Khon Kaen Province; 5, Koeng Subdistrict, Mueang District, Maha Sarakham Province; 6, Mueang District, Mukdahan Province; 7, Phu Ruea District, Loei Province; 9, Kut Bak District, Sakon Nakhon Province; and 10, Khao Yai, Nakhon Ratchasima Province. Sample numbers correspond to those listed in Table 1.

**Fig. 2:**
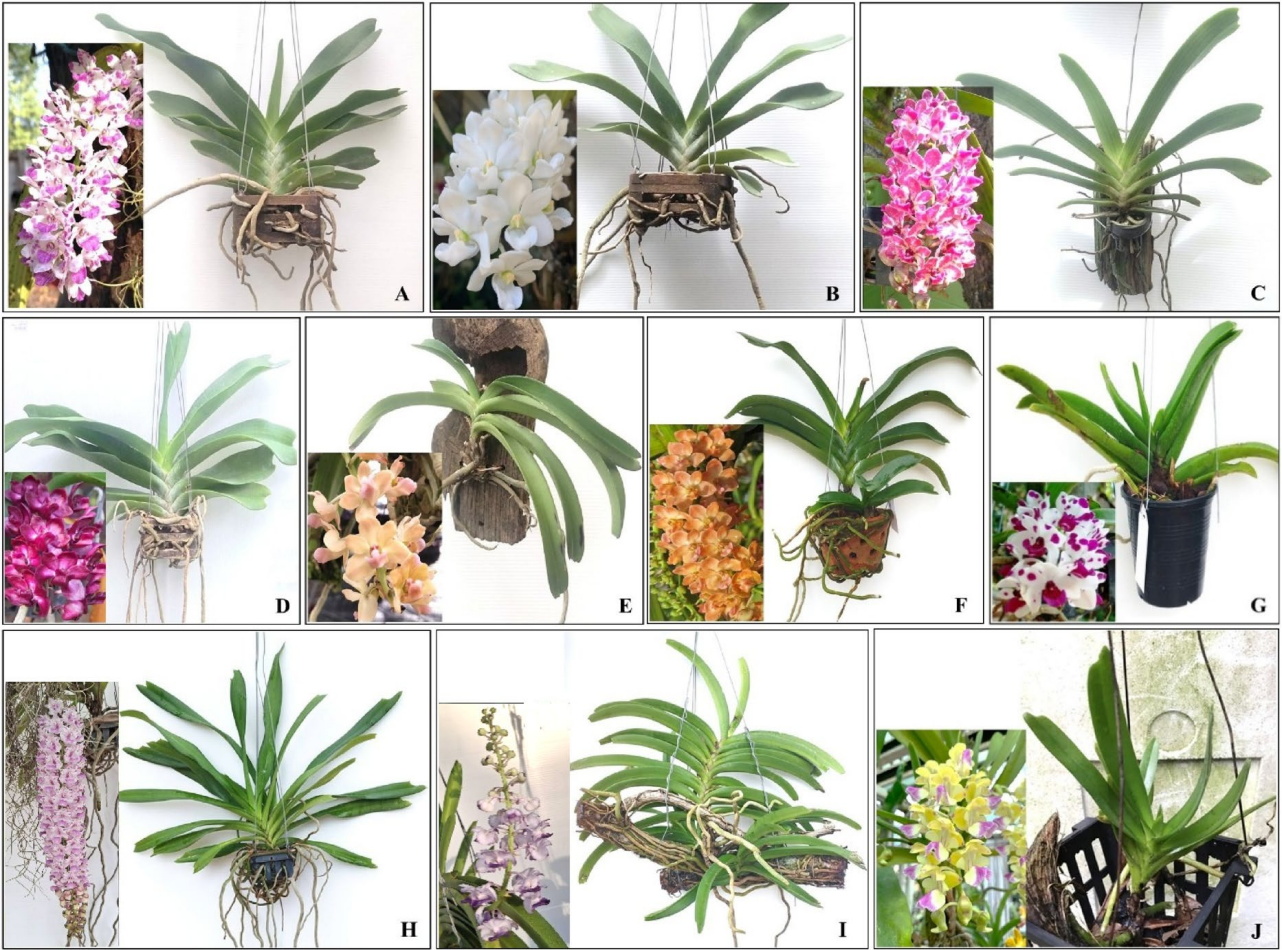
Representative morphological features of the sampled orchid accessions used in this study. Images illustrate rhizome, leaf, and floral traits, including flower coloration, labellum shape, and inflorescence structure, recorded prior to molecular and phytochemical analyses. The taxa shown are: **A** *R. gigantea*, **B** *R. gigantea* var. *harrisonianum*, **C** *R. gigantea* ‘Chang Phlai’, **D** *R. gigantea* var. *rubrum*, **E** *R. gigantea* var. *vivaphandhul*, **F** *R. gigantea* ‘Ruxaporn’, **G** *R. gigantea* ‘Kultana’, **H** *R. retusa*, **I** *R. coelestis*, and **J** *A. houlettiana*.

This study was conducted at the Laboratory of Molecular Genetics, Department of Biology, Faculty of Science, Mahasarakham University, Thailand. Nine *Rhynchostylis* accessions representing three species were collected from various locations in northeastern Thailand, encompassing both wild habitats and cultivated areas. In addition, *Aerides houlletiana* was selected as the outgroup in this study as a representative of a closely related genus to *Rhynchostylis* within the subtribe Aeridinae. Taxonomic identification was undertaken by J. Saengprajak, and voucher specimens were deposited in the Herbarium of the Faculty of Science, Mahasarakham University, Thailand. Notably, *R. gigantea* was observed growing naturally on tamarind trees at Wat Pa Mancha Khiri, Mancha Khiri District, Khon Kaen Province, where representative specimens were collected. The sampling map was prepared using QGIS software, with base map layers derived from Natural Earth (public domain) and OpenStreetMap contributors (ODbL license). Morphological characteristics, taxonomic identifications, distribution, collection sites, and voucher numbers are summarized in Table 1 and illustrated in Figs. 1 and 2. Wild-collected materials of *R. gigantea* and *R. retusa* were obtained with permission from the Plant Varieties Protection Office, Department of Agriculture, Thailand, in compliance with the IUCN Policy Statement on Research Involving Species at Risk of Extinction and CITES.

**Table 1:** Collection sites, accession details, and floral characteristics of *Rhynchostylis* species and *Aerides houlettiana* from Northeast Thailand.

S. No.	Location (Province)	GPS Coordinates	Altitude (m)	Species collected	Voucher Specimen	Source (Thai name)	Distinctive floral characteristics
1	Wat Pa Mancha Khiri, Mancha Khiri, Khon Kaen	16°07’07.2"N 102°32’00.8"E	163.16	*Rhynchostylis* *gigantea* var. *gigantea* (Lindl.) Ridl.	MSUT-8771	Wild (Chang Kra)	White flowers with red-pink spots
2	Khon Kaen University, Khon Kaen	16°27’52.9"N 102°48’47.0"E	176.11	*Rhynchostylis gigantea* var. *harrisonianum* Holtt.	MSUT-8778	Cultivated (Chang Pheuk)	Pure white flowers
3	Khon Kaen University, Khon Kaen	16°27’52.9"N 102°48’47.0"E	176.11	*Rhynchostylis gigantea* ‘Chang Phlai’	MSUT-8777	Cultivated (Chang Phlai)	White flowers with broad reddish-pink blotches
4	Mancha Khiri, Khon Kaen	16°07’28.6"N 102°32’20.9"E	163.16	*Rhynchostylis gigantea* var. *rubrum* Sagarik	MSUT-8776	Cultivated (Chang Daeng)	Pure dark-red flowers
5	Koeng Subdistrict, Mueang, Maha Sarakham	16°12’50.1"N 103°16’34.2"E	143.98	*Rhynchostylis gigantea* var. *vivaphandhul*	MSUT-8775	Cultivated (Chang Preme-Peach)	Bright orange, peach, and soft pink flowers; broad outward-curved labellum
6	Mueang, Mukdahan	16°30’31.8"N 104°43’43.7"E	160.8	*Rhynchostylis gigantea* ‘Ruxaporn’	MSUT-8780	Cultivated (Chang Som), Hybrid (*R. gigantea* var. *harrisonianum* × *R. gigantea* var. *rubrum*)	Pale grayish-orange flowers
7	Phu Ruae, Loei	17°27’7.7"N 101°21’41.8"E	624.0	*Rhynchostylis gigantea* ‘Kultana’	MSUT-8779	Cultivated (Chang Cartoon), Hybrid (*R. gigantea* var. *harrisonianum* × *R. gigantea* var. *rubrum*)	White flowers with reddish-purple blotches
8	Khon Kaen University, Khon Kaen	16°27’52.9"N 102°48’47.0"E	176.11	*Rhynchostylis retusa* (L.) Blume	MSUT-8772	Wild (Ai-raet)	White to pale pink flowers with bright purple or magenta spots
9	Kut Bak, Sakon Nakhon	17°04’18.5"N 103°49’04.9"E	204.92	*Rhynchostylis coelestis* (Rchb.f.) A.H.Kent	MSUT-8773	Cultivated (Khao Kae)	Pale blue to light lavender flowers
10	Khao Yai, Nakhon Ratchasima	14°52’13"N 102°00’22"E	227.03	*Aerides houlletiana* Rchb.f.	MSUT-8774	Cultivated (Ueang Kulap Luang Korat)	Creamy white to pale yellow flowers with reddish-purple to magenta spots

### Genomic DNA extraction, PCR amplification of DNA barcoding regions and sequencing

Leaf samples were finely ground in liquid nitrogen, and genomic DNA was extracted using the PureDireX Genomic DNA Isolation Kit for Plants (Thermo Fisher Scientific Co., Ltd.), following the manufacturer’s protocol. The quality of the extracted DNA was assessed by electrophoresis on a 1.0% agarose gel prepared in 1× TBE buffer and visualized using a GelDoc Go Gel Imaging System (Bio-Rad, USA), alongside a 100 bp DNA ladder RTU (GeneDirex, Inc.). DNA bands were visualized with ViSafe Green Gel Stain (Vivantis^®^, Malaysia). DNA concentration and purity were quantified using a NanoDrop Spectrophotometer (NND-1 NDL-PLUS-GL, Thermo Fisher Scientific Co., Ltd., USA). The DNA was then diluted to a working concentration of 50 ng/µL with nuclease-free water (Invitrogen™, Thermo Fisher Scientific Co., Ltd.) and stored at −20 °C to preserve DNA integrity, with samples processed within an appropriate time frame.

Amplification of the DNA barcode regions-internal transcribed spacer (ITS) and *maturase K* (*matK*) was performed using a PCR thermal cycler (Biometra TAdvanced, Bio-Active Co., Ltd.). Primer sequences, PCR reaction components, and thermal cycling conditions are detailed in Table 2. PCR products were analyzed by electrophoresis on a 1.0% agarose gel as previously described. Non-template controls were included in all reactions to ensure the absence of contamination and to validate primer specificity. PCR amplicons of approximately 500 bp (ITS) and 800–1000 bp (*matK*) were subjected to bidirectional Sanger sequencing, performed in triplicate for each sample using the original amplification primers to minimize sequencing artifacts. Sequencing was conducted by U2Bio (Thailand) Co., Ltd. using an ABI 3730XL DNA Analyzer (South Korea). Raw sequence reads were aligned and edited using BioEdit software, with non-aligned regions and ambiguous ends trimmed to minimize missing data. Consensus sequences were generated by assembling the forward and reverse reads, and their identities were confirmed using the Basic Local Alignment Search Tool (BLASTn) available on the NCBI website (https://blast.ncbi.nlm.nih.gov/Blast.cgi).

**Table 2:** Primer details, PCR reaction components, and thermocycling conditions used for amplification of the *matK* and ITS regions in *Rhynchostylis* and related taxa.

Target region	Primer sequence (5´- 3´)	Reaction mixture (Total volume = 25 µL)	PCR protocol	Reference
*matK*	Forward: 5′-CGATCTATTCATTCAATATTTC-3′ Reverse: 5′-TCTAGCACACGAAAGTCGAAGT-3′	The PCR mixture contained 1.2 mM MgCl₂, 1X PCR buffer, 0.8 mM dNTPs, 0.02 µM each forward and reward primer, 1 U nano*Taq* hot-start DNA polymerase, and 50–100 ng genomic DNA, with the final volume adjusted using nuclease-free water.	Pre-denaturation (1 cycle) 94 °C for 3 min Denaturation, Annealing, Extension (35 cycles) 94 °C for 30 s 42 °C for 48 s 72 °C for 1 min Final Extension (1 cycle) 72 °C for 10 min	^43^
ITS	Forward: 5´-CCTTATCATTTAGAGGAAGGA-3´ Reverse: 5´-TCCTCCGCTTATTGATATGC-3´	PCR reactions consisted of 2 mM MgCl₂, 1X PCR buffer, 1.5 mM dNTPs, 0.05 µM each forward and reward primer, 1.5 U nano*Taq* hot-start DNA polymerase, and 50–100 ng genomic DNA, with the final volume adjusted using nuclease-free water.	Pre-denaturation (1 cycle) 94 °C for 1.5 min Denaturation, Annealing, Extension (33 cycles) 94 °C for 1 min 55 °C for 1 min 72 °C for 1 min Final Extension (1 cycle) 72 °C for 4 min	^44^

All PCR reagents were sourced from BioHelix Co., Ltd., Taiwan. The differences in PCR cycling parameters between the *matK* and ITS reactions reflect the optimization required for each locus and were not due to the use of different reagents or kits.

### Sequence analysis and genetic relationship analysis

The homology of ITS and *matK* sequences was assessed using the BLASTn tool with default parameters. A sequence identification was considered accurate when the top BLAST hit corresponded to the expected species or genus. Conversely, if the best match did not align with the anticipated species, genus, or family, the identification was classified as ambiguous. Nucleotide sequences obtained from the ITS and *matK* regions were trimmed at both termini to remove low-quality or ambiguous bases. The final consensus sequences were deposited in the GenBank database (NCBI, USA) under the assigned accession numbers. Sequence similarity (% Match) was calculated as the proportion of matched base pairs to the total aligned length, following the method described by Altschul et al. (1997)^37^. Sequence coverage (% Coverage) was determined as the ratio of the total query length to the aligned length, based on the approach outlined by Madden (2013)^38^. Furthermore, the standard error (SE) of the match proportion was estimated using the binomial standard error formula described by Newcombe (1998)^39^. These values are presented in Table 3.

Multiple sequence alignments were produced using Clustal Omega (https://www.ebi.ac.uk/Tools/msa/clustalo/). Phylogenetic analyses of the ITS and *matK* datasets were performed in MEGA11^40^ using the maximum likelihood (ML) method. The best-fit nucleotide substitution model (Kimura 2-parameter; K2P) was selected according to the Bayesian Information Criterion (BIC) implemented in MEGA11 and applied to ML tree reconstruction. Neighbour-joining (NJ) analyses were also conducted based on pairwise genetic distances. Nodal support was evaluated using 1,000 bootstrap replicates. Bootstrap support (BS) values were interpreted following Kress et al. (2002): strong (> 85%), moderate (70–85%), weak (50–69%), and poor (< 50%)^41^. Branch lengths represent the expected number of nucleotide substitutions per site and therefore provide a measure of genetic divergence among taxa^42^. *A. houlletiana* was selected as the outgroup because it is phylogenetically close to, but distinct from, the *Rhynchostylis* clade within the subtribe Aeridinae. The resulting alignments were subsequently inspected and curated to improve alignment quality. Poorly aligned regions were removed prior to phylogenetic analyses. Gap positions were treated as missing data, and ambiguous sites were excluded when they could not be confidently resolved. The ITS and *matK* datasets were analyzed separately to allow comparison between nuclear and plastid phylogenetic signals, as incongruence between these markers may reflect hybridization or lineage sorting in orchids.

### Quantification of total phenolic and flavonoid contents

Orchid leaf samples were used for the extraction of bioactive compounds. The samples were oven-dried at 45 °C, ground into a fine powder, and stored at −20 °C until analysis. Extraction was performed following a modified protocol adapted from Zhang et al. (2007)^45^, in which 2 g of powdered sample was mixed with 20 mL of 80% ethanol and agitated at 250 rpm for 24 h. The mixture was then centrifuged at 10,000 rpm for 15 min, filtered through Whatman No. 0.1 filter paper, and the solvent was evaporated at 45 °C using a rotary evaporator. The resulting crude extract was stored at −20 °C (Panasonic, Thailand) prior to analysis, with samples processed within an appropriate time frame.

TPC was determined using a modified version of the Folin-Ciocalteu method described by Radošević et al. (2017) and Thomas et al. (2018)^46,47^. In a 96-well microplate, 20 µL of crude extract (20 mg/mL) was combined with 100 µL of 10% Folin-Ciocalteu reagent, followed by 80 µL of 7.5% sodium bicarbonate (NaHCO₃). After a 90-minute incubation in the dark, absorbance was measured at 750 nm using a microplate reader. TPC was quantified using a gallic acid standard curve and expressed as milligrams of gallic acid equivalents per gram of dry weight (mg GAE/g DW). Measurements were conducted in triplicate for each independently prepared extract, and the data were used for statistical analysis.

TFC was determined following a modified protocol from Tian et al. (2016)^48^. In a 96-well plate, 20 µL of crude extract (20 mg/mL) was mixed with 60 µL of distilled water and 10 µL of 5% sodium nitrite (NaNO₂). After a 6-minute incubation at room temperature, 10 µL of 10% aluminum chloride (AlCl₃•6 H₂O) was added, followed by a 5-minute incubation. Then, 100 µL of 1 M sodium hydroxide (NaOH) was added, and the mixture was incubated for an additional 12 min. Absorbance was measured at 510 nm. TFC was calculated using a rutin standard curve and expressed as milligrams of rutin equivalents per gram of dry weight (mg RE/g DW). All measurements were performed in triplicate for each independently prepared extract, and the data were used for statistical analysis.

### Antioxidant activity assay

Antioxidant activity was assessed using the DPPH radical scavenging assay, following a modified protocol from Zhang et al. (2019)^49^. A 0.2 mM DPPH solution was prepared in methanol. In a 96-well microplate, 20 µL of crude extract (20 mg/mL) was combined with 180 µL of the DPPH solution. The mixture was incubated in the dark at room temperature for 30 min. A control well containing 180 µL of DPPH solution without extract was included to establish baseline absorbance. Following incubation, absorbance was measured at 517 nm using a microplate reader. Antioxidant capacity was expressed as milligrams of Trolox equivalent antioxidant capacity per gram of dry weight (mg TEAC/g DW). All assays were conducted in triplicate technical replicates for each independently prepared extract and analyzed statistically.

### Statistical analysis

One-way analysis of variance (ANOVA) was conducted using SPSS Statistics software (Version 15.0 for Windows) to evaluate differences in TPC, TFC, and antioxidant activities of the ethanolic extracts. Statistical significance was assessed at the 95% confidence level (*p* < 0.05), and mean separation was performed using Duncan’s multiple range test^50^.

## Results

### Molecular analysis of barcode sequences

**Fig. 3:**
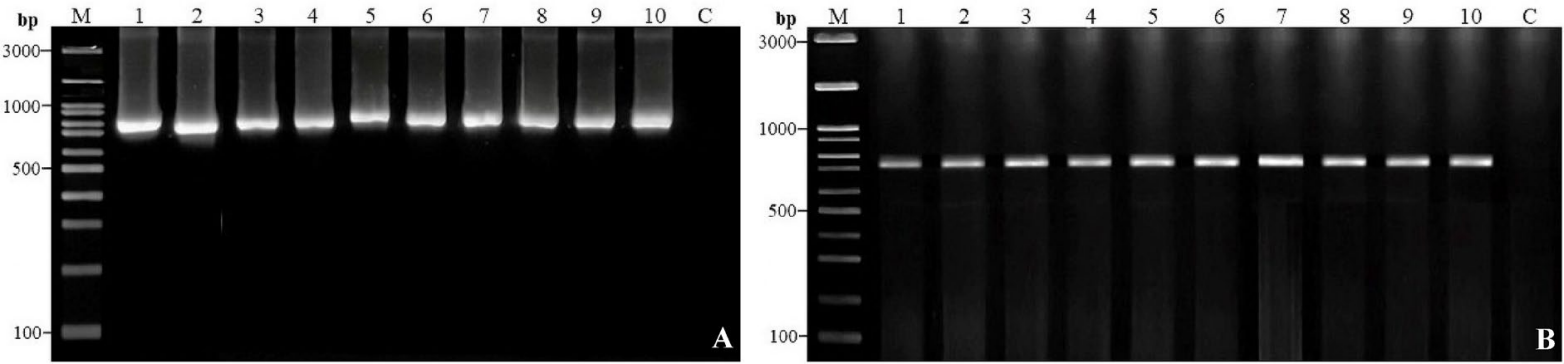
PCR amplification profiles of the ITS region (**A**) and the *matK* gene (**B**) from the sampled orchid accessions. Lane M: 100 bp DNA ladder; lanes 1–10 correspond to the accessions listed in Table 1; C: negative control.

The ITS and *matK* regions were successfully amplified and sequenced for all sampled accessions and were used as standard DNA barcode loci to assess genetic relationships among *Rhynchostylis* accessions and one *Aerides* accession. PCR amplification produced clear bands of the expected sizes for all samples (Fig. 3). Replicate PCR experiments and full-length gel images are provided in the Supplementary Information. Following PCR amplification and sequencing, the trimmed sequence lengths ranged from approximately 497–691 bp for ITS and 113–416 bp for *matK*. The sequences were analyzed using the BLASTn tool to identify their closest matches among reference sequences in the GenBank database. Most sequences showed very high similarity to their closest GenBank matches, often exceeding 90%, with consistently complete coverage (100%) indicating full alignment with the reference sequences. Moreover, the standard error (SE) values were very low (0.0000–0.0191), demonstrating high precision in the similarity estimates. Polymorphic sites were detected in both the ITS and *matK* regions across the analyzed accessions, demonstrating their effectiveness for species-level discrimination, as further supported by estimates of average evolutionary divergence among species presented in Table 4 (full data not shown). The datasets generated and analyzed during the current study are available in the National Center for Biotechnology Information (NCBI) repository, with hyperlinks provided under each accession number in Table 3.

BLAST analysis of the ITS sequences revealed that *R. gigantea* and its varieties including *R. gigantea* ‘Chang Phlai’, *R. gigantea* var. *rubrum*, *R. gigantea* var. *vivaphandhul*, and cultivated forms including ‘Chang Phlai’, ‘Ruxaporn’, and ‘Kultana’ all shared high sequence similarity with previously reported *R. gigantea* accessions. Notably, *R. gigantea* ‘Chang Phlai’ closely matched *Rhynchostylis* sp. (accession no. PQ289154), while *R. gigantea* var. *rubrum* and *R. retusa* displayed strong similarity to *R. retusa* accessions. In contrast, ITS sequences of *R. gigantea* var. *harrisonianum* aligned closely with *R. gigantea* (EF670366) but exhibited some divergence from the typical *R. gigantea*. The ITS sequence of *R. coelestis* showed high similarity to *R. coelestis* voucher KFBG2606, while *A. houlettiana* matched closely with *A. falcata* (JX202646).

For the *matK* region, most *R. gigantea* samples exhibited near-perfect matches to *R. gigantea* accessions, such as vouchers KFBG3178 and KFBG2661. However, the *matK* sequences of *R. gigantea* var. *harrisonianum* and *R. gigantea* ‘Kultana’ showed closer similarity to *R. retusa* isolates than to typical *R. gigantea* entries. Additionally, *R. retusa* matched well with *R. retusa* voucher JK-DEBCR, *R. coelestis* aligned closely with voucher Lior124, and *A. houlettiana* matched *A. houlettiana* voucher KFBG2575A.

Overall, the results demonstrated that both ITS and *matK* regions are effective molecular markers for distinguishing among accessions within *Rhynchostylis* and for differentiating *Rhynchostylis* from closely related genera. However, some discrepancies, such as the clustering of *Aerides houlletiana* within *Rhynchostylis*, indicate that these markers alone may have limitations in fully resolving intergeneric relationships, although minor differences in sequence similarity were observed between the two barcode regions (Table 3).

**Table 3:** Sequence similarity results based on ITS and *matK* regions, showing the closest matches to *Rhynchostylis* and *Aerides* species retrieved from the NCBI database.

Sample	Regions	Accession number	Closest Match (GenBank)	Match Accession No.	Aligned Length (bp)	Similarity	% Match	Coverage (%)	SE
*R. gigantea*	ITS	PQ289154	*R. gigantea*	KY966664	497	431/497	94.3	100	0.0090
	*matK*	PQ329541	*R. gigantea*	EF655778	303	303/303	91.0	100	0.0140
*R. gigantea* var. *harrisonianum*	ITS	PQ308354	*R. gigantea*	EF670366	691	607/691	87.9	100	0.0143
	*matK*	PQ365689	*R. retusa*	MK607423	149	140/149	100.0	100	0.0000
*R. gigantea *‘Chang Phlai’	ITS	PQ308351	*Rhynchostylis* sp.	PQ289154	186	167/186	88.2	100	0.0140
	*matK*	PQ329542	*R. gigantea*	KY966950	300	300/300	99.4	100	0.0043
*R. gigantea* var. *rubrum*	ITS	PQ308350	*R. retusa*	MK483276	687	666/687	92.1	100	0.0134
	*matK*	PQ360717	*R. gigantea*	EF655778	416	415/416	98.8	100	0.0060
*R. gigantea* var. *vivaphandhul*	ITS	PQ319762	*R. gigantea*	DQ091717	688	649/688	96.1	100	0.0076
	*matK*	PQ360718	*R. gigantea*	EF655778	413	376/413	93.7	100	0.0191
*R. gigantea* ‘Ruxaporn’	ITS	PQ345388	*R. gigantea*	KY966665	536	471/536	95.5	100	0.0082
	*matK*	PQ360719	*R. gigantea*	KY966948	113	113/113	95.5	100	0.0144
*R. gigantea* ‘Kultana’	ITS	PQ345416	*R. gigantea*	KY966664	525	463/525	94.3	100	0.0090
	*matK*	PQ360720	*R. retusa*	EU558938	311	309/311	91.0	100	0.0140
*R. retusa*	ITS	PQ308353	*R. retusa*	MW475276	403	371/403	87.9	100	0.0143
	*matK*	PQ360716	*R. retusa*	MK607423	330	326/330	100.0	100	0.0000
*R. coelestis*	ITS	PQ308352	*R. coelestis*	KY966663	649	624/649	88.2	100	0.0140
	*matK*	PQ360715	*R. coelestis*	NC_086850	159	149/159	99.4	100	0.0043
*A. houlettiana*	ITS	PQ319759	*A. falcata*	JX202646	641	612/641	92.1	100	0.0134
	*matK*	PQ360714	*R. gigantea*	KY966705	224	214/224	98.8	100	0.0060

### Estimation of sequence divergence

Sequence divergence among the analyzed regions is presented in Table 5. The divergence values for the ITS and *matK* regions ranged from 0.117 to 0.695 and from 0.015 to 1.917, respectively. Within the ITS region, intraspecific divergence varied from 0.117 to 0.695, whereas the *matK* region exhibited a broader range of intraspecific variation, spanning from 0.015 to 1.917.

Interspecific divergence values ranged from 0.177 to 0.268 for the ITS region and from 0.741 to 1.381 for the *matK* region. The mean intraspecific distances were calculated as 0.301 for ITS and 1.109 for *matK*, while the mean interspecific distances were slightly higher, at 0.208 for ITS and 1.137 for *matK*.

**Table 4:** Estimates of average evolutionary divergence among species based on ITS and *matK* sequences.

Parameter	ITS	*matK*
Range of intraspecific distance	0.117–0.695	0.015–1.917
Range of interspecific distance	0.177–0.268	0.741–1.381
Mean intraspecific distance	0.301	1.109
Mean interspecific distance	0.208	1.137

All ambiguous positions were removed for each sequence pair prior to analysis. Evolutionary divergence estimates were calculated using the Kimura 2-parameter model implemented in MEGA version 11.

Base substitution patterns in the ITS and *matK* regions were evaluated across all codon positions (1st, 2nd, and 3rd nucleotides) using the Tamura-Nei model (1993)^51^, as presented in Table 6. In the ITS region, the highest rates of transitional substitutions were observed for A to G (13.33%) and T to C (15.91%), while in the *matK* region, high transitional rates were found for C to T (17.61%) and G to A (17.75%). In both regions, the frequency of transitional substitutions exceeded that of transversional substitutions, with particularly elevated rates of C to T and G to A transitions in *matK*.

To evaluate interspecific and intraspecific divergence, both the range and mean values were considered. The results indicated that the *matK* region exhibited greater intraspecific variation than the ITS region, as reflected by its broader range and higher mean intraspecific distance. In contrast, the ITS region demonstrated slightly higher interspecific divergence compared to *matK*, suggesting that the ITS region may offer greater resolution for species differentiation.

**Table 5:** Nucleotide substitution patterns (%) in the ITS and *matK* regions.

From/To	ITS				*matK*			
	A	T	C	G	A	T	C	G
A	-	*5.09*	*7.14*	**13.33**	-	*8.46*	*3.8*	**7.72**
T	*5.07*	-	**15.91**	*8.38*	*8.54*	-	**7.91**	*3.71*
C	*5.07*	**11.34**	-	*8.38*	*8.54*	**17.61**	-	*3.71*
G	**8.07**	*5.09*	*7.14*	-	**17.75**	*8.46*	*3.8*	-

Substitution patterns and rates were estimated using the Tamura-Nei model (1993). Rates of different transitional substitutions are shown in bold and those of transversionsal substitutions are shown in italics.

### Phylogenetic analysis of *Rhynchostylis* and related species

#### ITS region

**Fig. 4:**
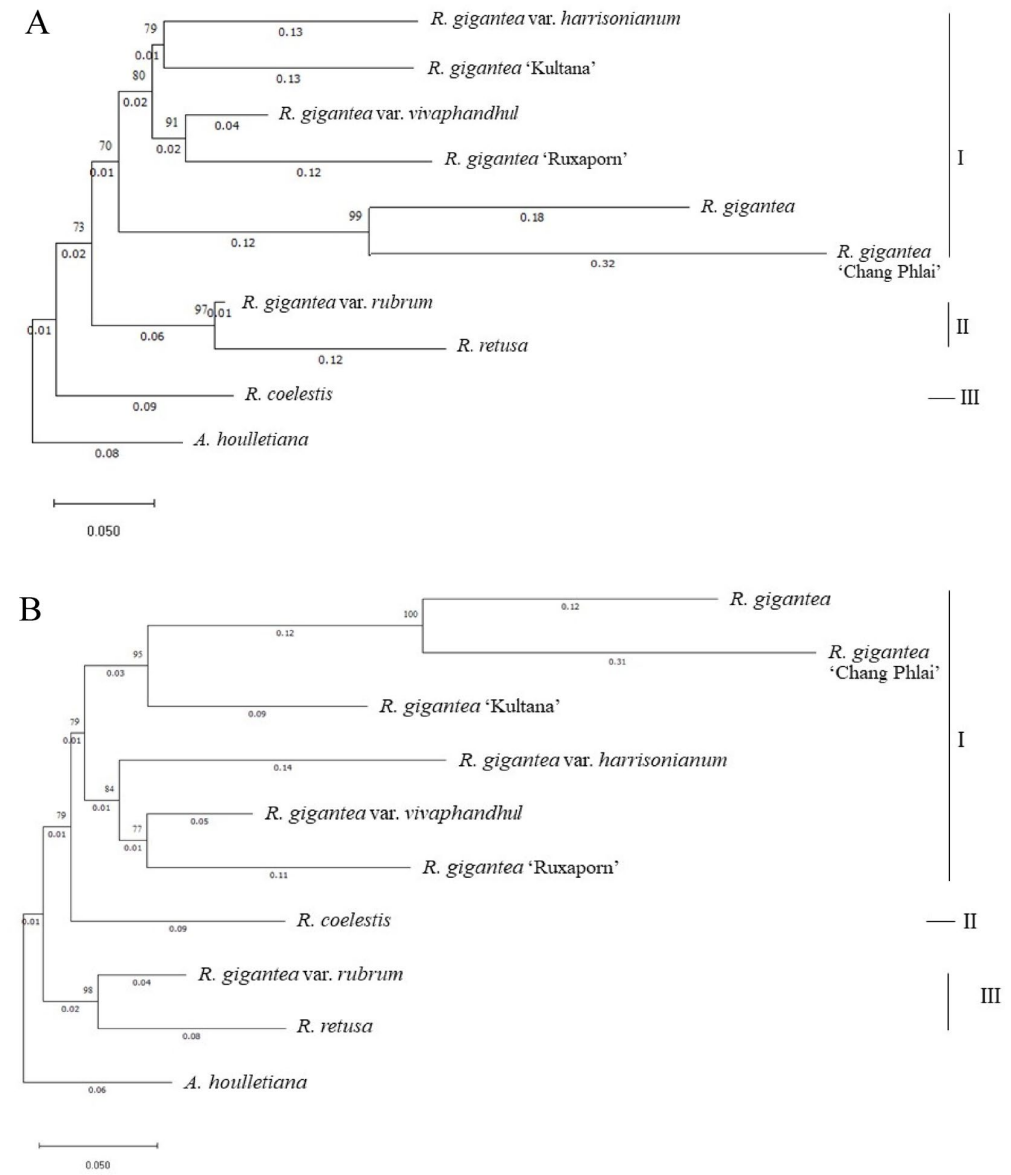
Phylogenetic trees reconstructed from ITS sequences using **A** maximum likelihood (ML) and **B** Neighbour-joining (NJ) methods. Bootstrap support values (1,000 replicates) are shown at the nodes. *A. houlletiana* was used as the outgroup. Branch lengths represent substitutions per site.

Maximum likelihood (ML) and Neighbour-joining (NJ) phylogenies based on ITS sequences consistently resolved two major lineages within the dataset (Fig. 4). *A. houlletiana* was recovered as a distinct outgroup with strong branch separation, confirming its evolutionary placement outside the *Rhynchostylis* clade. *R. retusa* and *R. coelestis* tended to form recognizable lineages distinct from most *R. gigantea* accessions, although their placement varied slightly depending on the marker and analytical method.

All *R. gigantea* accessions formed a single major lineage that contained several distinct but closely related subclades. A strongly supported subgroup comprised *R. gigantea* var. *harrisonianum*, *R. gigantea* ‘Kultana’, *R. gigantea* var. *vivaphandhul*, and *R. gigantea* ‘Ruxaporn’, indicating a high degree of sequence similarity among these cultivated forms. *R. gigantea* and *R. gigantea* ‘Chang Phlai’ clustered together as a closely related sister subgroup, whereas *R. gigantea* var. *rubrum* showed slight separation from the core *R. gigantea* lineage, suggesting moderate divergence. Overall, the topologies obtained from ML and NJ were highly congruent, with ML yielding generally higher bootstrap support across internal nodes.

#### *matK* region

**Fig. 5:**
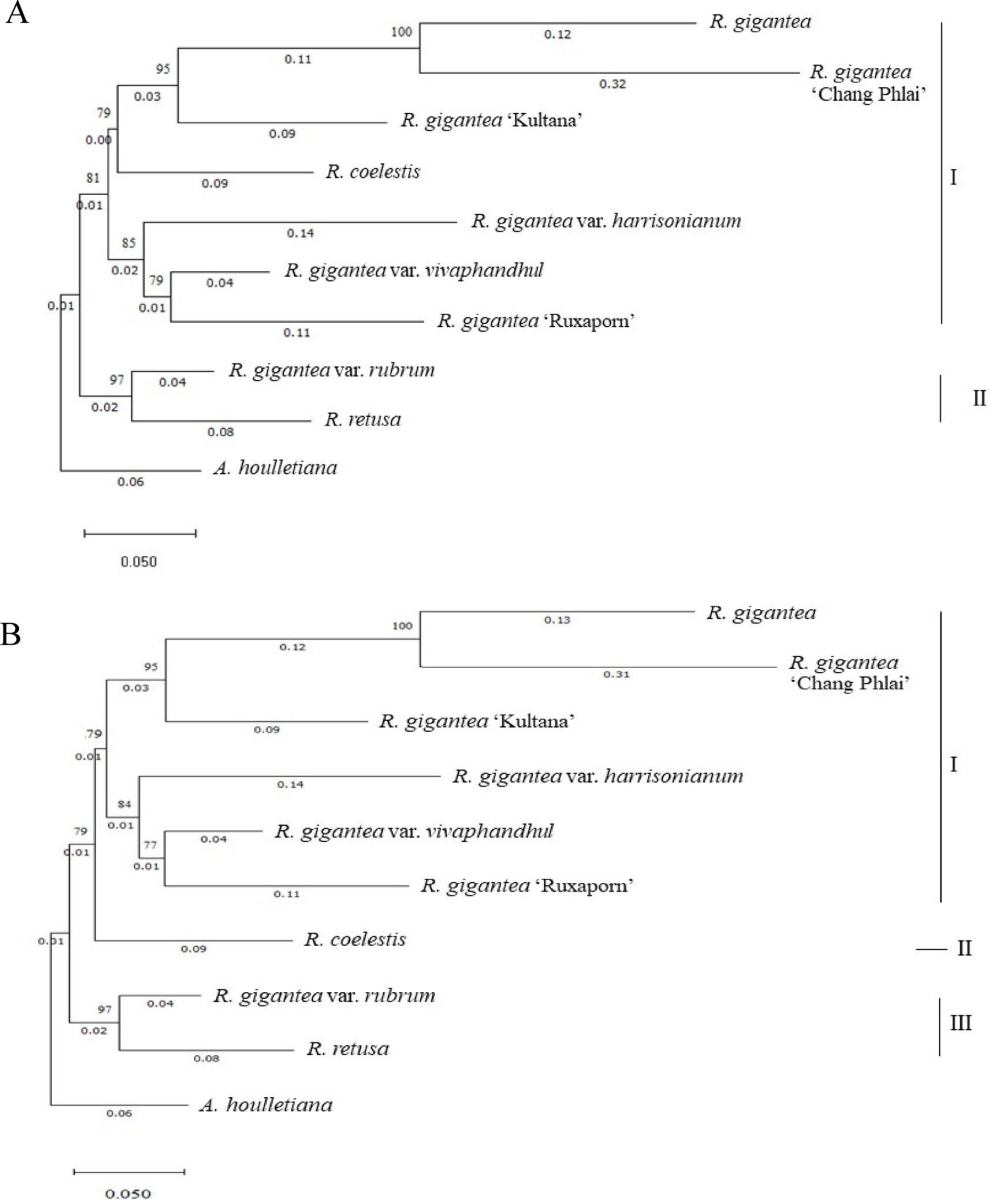
Phylogenetic trees reconstructed from *matK* sequences using **A** maximum likelihood (ML) and **B** Neighbour-joining (NJ) methods. Bootstrap support values (1,000 replicates) are shown at the nodes. *A. houlletiana* was used as the outgroup. Branch lengths represent substitutions per site.

Phylogenetic analysis of the *matK* region produced a broadly similar topology to that of ITS, with *A. houlletiana* again indicating differentiation of *R. retusa* and *R. coelestis*, although their relationships with the R. gigantea complex were not identical across markers (Fig. 5). Within *R. gigantea*, the ‘Kultana’ and ‘Ruxaporn’ groups consistently formed a strongly supported sister clade in both ML and NJ analyses, indicating close genetic affinity and a probable shared hybrid origin. These hybrid forms were placed adjacent to, but distinct from, wild-type *R. gigantea* and *R. gigantea* var. *harrisonianum*, which together formed a Neighbouring clade.

In contrast to the ITS tree, *R. gigantea* var. *vivaphandhul*, *R. gigantea* var. *rubrum*, and *R. retusa* clustered within the same broader lineage, indicating slight differences in phylogenetic resolution between plastid and nuclear markers. As with ITS, ML analysis of *matK* sequences produced higher bootstrap support than NJ for most nodes.

#### Comparison between markers

Both ITS and *matK* regions provided consistent species-level resolution, generally distinguishing *R. retusa* and *R. coelestis* from most *R. gigantea* forms and confirming the distinct placement of *A. houlletiana* as an outgroup. However, the ITS region exhibited greater discriminatory power among closely related *R. gigantea* forms, whereas *matK* produced slightly broader clustering patterns. Taken together, these findings indicate that ITS and *matK* provide complementary phylogenetic signals that reliably support species delimitation within *Rhynchostylis*. Bootstrap values ≥ 70% were interpreted as indicating strong node support.

### Polyphenol content analysis

**Fig. 6:**
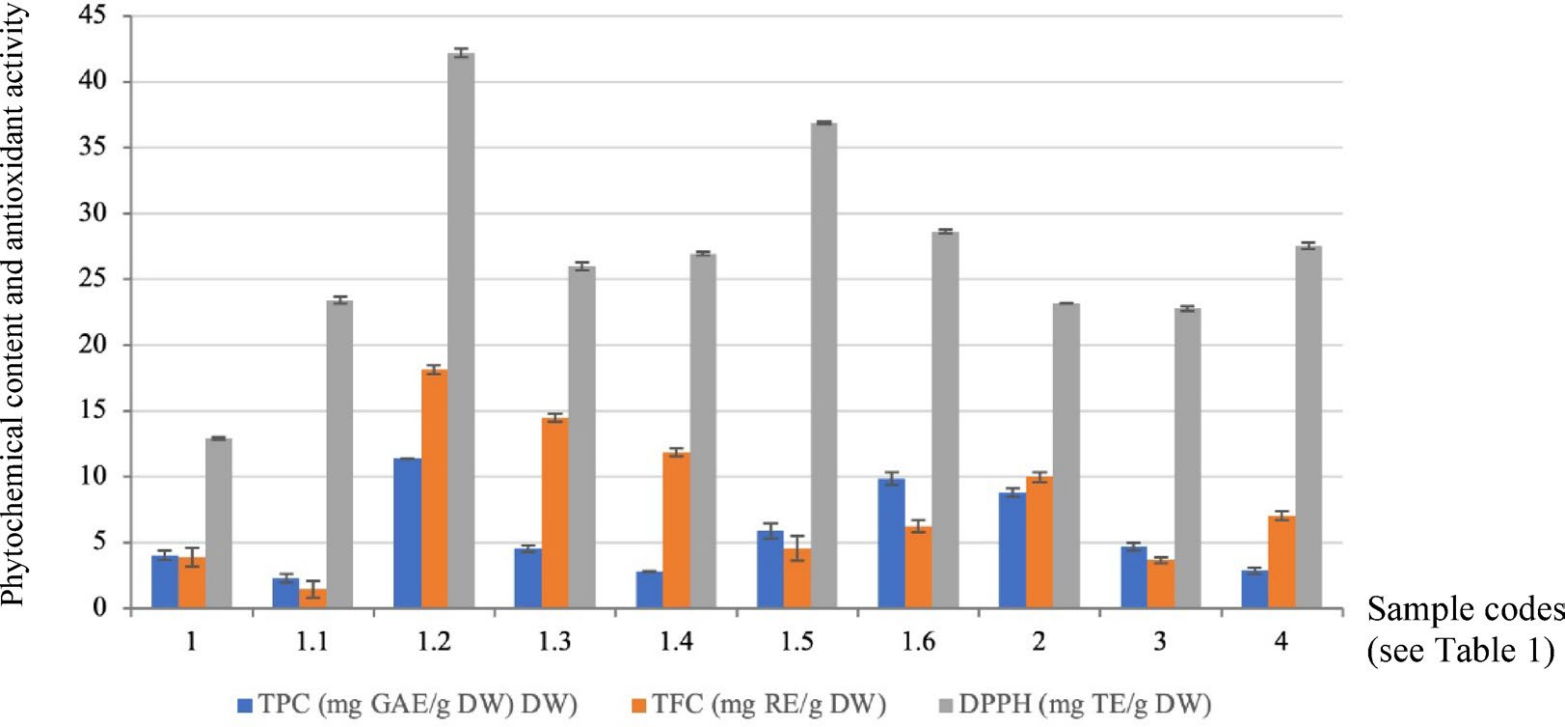
Comparison of total phenolic content (TPC; mg GAE g⁻¹ DW), total flavonoid content (TFC; mg RE g⁻¹ DW), and antioxidant activity (DPPH radical scavenging; mg TE g⁻¹ DW) among the sampled orchid accessions. Error bars represent the standard error (SE) of triplicate technical measurements. Accessions are arranged according to a hierarchical coding scheme in which *R. gigantea* is designated as 1, with its varieties and cultivated forms indicated by decimal subcodes: var. *harrisonianum* (1.1), ‘Chang Phlai’ (1.2), var. *rubrum* (1.3), var. *vivaphandhul* (1.4), ‘Ruxaporn’ (1.5), and ‘Kultana’ (1.6). Other taxa are numbered sequentially as *R. retusa* (2), *R. coelestis* (3), and *Aerides houlletiana* (4).

The TPC and TFC of ethanolic extracts from *Rhynchostylis* and related species are summarized in Table 6; Fig. 6. The highest TPC was observed in *R. gigantea* ‘Chang Phlai’ (11.38 ± 0.01 mg GAE/g DW), which was significantly higher than in the other taxa. This was followed by *R. gigantea* ‘Kultana’ (9.87 ± 0.49 mg GAE/g DW) and *R. retusa* (8.79 ± 0.34 mg GAE/g DW), both of which exhibited intermediate TPC levels. In contrast, *R. gigantea* var. *harrisonianum* (2.30 ± 0.32 mg GAE/g DW) and *A. houlettiana* (2.88 ± 0.23 mg GAE/g DW) showed the lowest TPC values among the species studied.

TFC varied significantly across the species, ranging from 1.47 ± 0.62 to 18.14 ± 0.33 mg RE/g DW. The highest TFC was found in *R. gigantea* ‘Chang Phlai’ (18.14 ± 0.33 mg RE/g DW), which was significantly higher than the other taxa. Intermediate TFC values were observed in *R. gigantea* var. *rubrum* (14.47 ± 0.31 mg RE/g DW) and *R. gigantea* var. *vivaphandhul* (11.85 ± 0.31 mg RE/g DW). In contrast, *R. gigantea* var. *harrisonianum* exhibited the lowest TFC (1.47 ± 0.62 mg RE/g DW).

Among the species analyzed, *R. gigantea* ‘Chang Phlai’ exhibited the highest TPC and TFC. These results suggest that these taxa possess considerable potential for medicinal applications due to their elevated polyphenolic profiles.

### Antioxidant activity

The antioxidant activity, measured by DPPH radical scavenging capacity, varied among the species studied, ranging from 12.91 ± 0.09 mg TE/g DW to 42.17 ± 0.33 mg TE/g DW (Table 6; Fig. 6). The highest antioxidant activity was observed in *R. gigantea* ‘Chang Phlai’ (42.17 ± 0.33 mg TE/g DW), which was significantly higher than that of the other taxa. This was followed by *R. gigantea* ‘Ruxaporn’ (36.87 ± 0.10 mg TE/g DW) and *R. gigantea* ‘Kultana’ (28.63 ± 0.15 mg TE/g DW), both exhibiting moderately high antioxidant capacities. In contrast, the lowest antioxidant activity was detected in *R. gigantea* (12.91 ± 0.09 mg TE/g DW).

Overall, *R. gigantea* ‘Chang Phlai’ consistently demonstrated the highest TPC, flavonoid content, and antioxidant activity among the species analyzed. *R. gigantea* ‘Kultana’ and *R. retusa* also exhibited relatively high phenolic and antioxidant properties, indicating their potential as promising candidates for medicinal applications.

**Table 6:** The total phenolic content (TPC), total flavonoid content (TFC), and antioxidant activities (DPPH) of ethanolic extracts from *Rhynchostylis* and related species.

Sample	Codes	TPC (mg GAE/g DW)	TFC (mg RE/g DW)	DPPH (mg TE/g DW)
*R. gigantea*	1	4.03 ± 0.36^e^	3.85 ± 0.71^f^	12.91 ± 0.09^g^
*R. gigantea* var. *harrisonianum*	1.1	2.30 ± 0.32^f^	1.47 ± 0.62^g^	23.41 ± 0.25^f^
*R. gigantea* ‘Chang Phlai’	1.2	11.38 ± 0.01^a^	18.14 ± 0.33^a^	42.17 ± 0.33^a^
*R. gigantea* var. *rubrum*	1.3	4.55 ± 0.22^e^	14.47 ± 0.31^b^	25.98 ± 0.31^e^
*R. gigantea* var. *vivaphandhul*	1.4	2.81 ± 0.05^f^	11.85 ± 0.31^c^	26.95 ± 0.15^d^
*R. gigantea* ‘Ruxaporn’	1.5	5.87 ± 0.58^d^	4.55 ± 0.94^f^	36.87 ± 0.10^b^
*R. gigantea* ‘Kultana’	1.6	9.87 ± 0.49^b^	6.22 ± 0.47^e^	28.63 ± 0.15^c^
*R. retusa*	2	8.79 ± 0.34^c^	9.97 ± 0.38^d^	23.18 ± 0.05^f^
*R. coelestis*	3	4.68 ± 0.33^e^	3.65 ± 0.24^f^	22.79 ± 0.17^f^
*A. houlettiana*	4	2.88 ± 0.23^f^	7.06 ± 0.34^e^	27.56 ± 0.24^d^

*Values are presented as mean ± standard error (*n* = 3). Means for total phenolic content (TPC), total flavonoid content (TFC), and DPPH antioxidant activity followed by different superscript letters within the same column are significantly different according to Duncan’s multiple range test (*p* < 0.05)^50^. GAE = gallic acid equivalent; RE = Rutin equivalents; TE = Trolox equivalent.

**Table 7:** Comparison of total phenolic content (TPC), total flavonoid content (TFC), and antioxidant activity (DPPH) among *Rhynchostylis* taxa and related species.

Parameter	Ranking (from highest to lowest)
TPC (mg GAE/g DW)	1.2 > 1.6 > 2 > 1.5 > 1.3 > 3 > 1 > 1.4 > 4 > 1.1
TFC (mg RE/g DW)	1.2 > 1.3 > 1.4 > 2 > 1.6 > 1.5 > 3 > 1 > 4 > 1.1
DPPH (mg TE/g DW)	1.2 > 1.5 > 1.6 > 1.4 > 1.3 > 4 > 2 > 3 > 1.1 > 1

Sample numbering follows a hierarchical system in which *R. gigantea* is designated as 1, with its varieties and cultivated forms indicated by sub-numbers: var. *harrisonianum* (1.1), ‘Chang Phlai’ (1.2), var. *rubrum* (1.3), var. *vivaphandhul* (1.4), ‘Ruxaporn’ (1.5), and ‘Kultana’ (1.6). Other species are numbered sequentially as *R. retusa* (2), *R. coelestis* (3), and *Aerides houlletiana* (4).

The rankings derived from the phytochemical analyses are summarized in Table 7. Across all assays, sample 1.2 (*R. gigantea* ‘Chang Phlai’) consistently showed the highest total phenolic and flavonoid contents, as well as the strongest antioxidant activity. In contrast, sample 1.1 (var. *harrisonianum*) consistently ranked lowest across the three parameters. Other taxa displayed intermediate positions, with some variation among assays; for example, samples 1.5 and 1.6 showed relatively high antioxidant capacity, whereas species-level samples such as *R. retusa* (2) and *R. coelestis* (3) occupied moderate positions in the rankings. Despite minor shifts in the order among assays, the overall pattern suggests a positive relationship between TPC, TFC, and DPPH across the sampled taxa. These results indicate that phytochemical variation among *Rhynchostylis* forms is reflected consistently in both compositional and functional assays (Table 7).

## Discussion

Accurate identification of *Rhynchostylis* taxa remains challenging because key morphological traits are influenced by environmental conditions and developmental stage, and diagnostic floral characters are often short-lived or overlapping among taxa^1,52^. Consequently, species delimitation based on morphology alone is frequently uncertain. To address this limitation, this study employed the ITS and *matK* DNA barcoding markers to reconstruct phylogenetic relationships among *Rhynchostylis* and *Aerides* accessions, complemented by phytochemical profiling to provide additional biological context.

### Screening and evaluation of candidate DNA barcoding regions

Morphological similarity among *Rhynchostylis* taxa can lead to misidentification, which may affect conservation planning and utilization^1^. Given the listing of these orchids under CITES Appendix II^10^, reliable taxonomic discrimination remains important. DNA barcoding markers such as ITS, *matK*, and *rbcL*, together with several non-coding plastid spacers, are widely used for plant identification^53^, particularly in groups where morphological differentiation is limited. Compared with morphological and phytochemical traits, which may vary with environmental conditions, DNA barcoding provides a standardized molecular framework for species recognition^54^.

In the present study, both ITS and *matK* loci were successfully amplified and sequenced across all accessions, and BLAST searches yielded consistent species-level matches. Nevertheless, some discrepancies were observed. For example, *matK* sequences from var. *harrisonianum* and ‘Kultana’ showed greater similarity to *R. retusa*, whereas their ITS sequences aligned with *R. gigantea*. Such incongruence may reflect intraspecific variation or hybrid ancestry^55^. These results support the use of multiple loci for reliable identification in taxonomically complex groups. Similar inconsistencies between molecular databases have been reported in other taxa, including jewel orchids and *Chenopodium murale*, where BLAST and BOLD outputs diverged due to database limitations^56^. In this study, GenBank provided consistent matches, supporting its suitability for orchid barcoding analyses.

### Phylogenetic inference using DNA barcoding markers

Estimating nucleotide substitution patterns is important for interpreting genetic divergence in phylogenetic analyses. In the present study, both ITS and *matK* regions provided sufficient sequence variation to resolve relationships among *Rhynchostylis* taxa. Although a distinct barcoding gap was not detected, the divergence values fell within the range reported for orchid barcoding studies, supporting the suitability of these loci for taxonomic inference in this group^18,21–23,57^. Consistent with previous work, ITS showed higher discriminatory power among closely related taxa, whereas *matK* contributed broader phylogenetic structure^58^.

Maximum likelihood and Neighbour-joining analyses of ITS sequences resolved two principal evolutionary lineages. *A. houlletiana* formed a clearly separated outgroup, while *R. retusa* and *R. coelestis* were consistently recovered outside the *R. gigantea* complex (Fig. 4). Within *R. gigantea*, cultivated and varietal forms clustered tightly with strong node support, indicating high nuclear sequence similarity that may reflect shared breeding backgrounds or recent divergence^59^. The wild type (*R. gigantea*) and *R. gigantea* ‘Chang Phlai’ formed a closely related sister subgroup, whereas var. *rubrum* was positioned slightly apart from the core lineage, suggesting moderate differentiation.

The *matK*-based phylogeny recovered a broadly similar topology (Fig. 5), again separating *R. retusa* and *R. coelestis* and confirming the distinct placement of *A. houlletiana*. However, some incongruence between nuclear and plastid trees was observed^60^. The cultivated accessions ‘Kultana’ and ‘Ruxaporn’ formed a strongly supported sister pair, whereas var. *vivaphandhul*, var. *rubrum*, and *R. retusa* were resolved within a broader lineage. Such marker discordance is commonly attributed to processes such as hybridization, incomplete lineage sorting, or the uniparental inheritance of plastid genomes, all of which have been frequently documented in orchid phylogenetic studies^61,62^. Bootstrap support values were generally higher in maximum-likelihood trees, suggesting greater robustness of likelihood-based inference compared with distance-based approaches.

When interpreted alongside floral morphology (Table 1; Fig. 2), the molecular results reveal a coherent taxonomic pattern. Species with distinctive floral architecture, such as *R. retusa* and *R. coelestis*, were generally resolved as recognizable lineages, thereby supporting their current taxonomic recognition. In contrast, cultivated *R. gigantea* accessions exhibited overlapping floral pigmentation and labellum morphology, particularly in ‘Kultana’ and ‘Ruxaporn’, consistent with their molecular clustering and previously reported hybrid origins^63,64^. The variable placement of var. *rubrum* and var. *vivaphandhul* across markers may likewise reflect more complex lineage histories. Overall, this pattern suggests that morphological variation within cultivated forms may partly reflect horticultural selection acting on hybrid genetic backgrounds^65^. Because the sampled accessions originate from different localities and cultivation histories, part of the observed genetic variation may reflect geographic isolation or horticultural selection rather than species-level divergence. Disentangling these effects would require denser population sampling within species.

The phylogenetic structure recovered in this study is consistent with previous molecular analyses of Aeridinae. Earlier multilocus studies similarly resolved *Rhynchostylis* as a distinct lineage within the *Aerides*-*Vanda* alliance and separate from related genera such as *Aerides* and *Vanda*^13,66^. Recent plastome-based analyses have suggested that *Rhynchostylis coelestis* may be phylogenetically embedded within the genus *Vanda*, potentially supporting an alternative generic placement^67^. Such results highlight that genome-scale datasets may recover relationships not fully resolved by barcode loci alone. Our results also align with broader Aeridinae phylogenies in which nuclear and plastid markers recover stable generic clades^68^. This concordance supports the reliability of the ITS and *matK* markers and strengthens confidence in the phylogenetic relationships inferred here. Although broader sampling across the full geographic range of the genus could further refine deep evolutionary relationships, the present dataset was designed to represent regional diversity in Thailand and to address species delimitation within the locally important *R. gigantea* complex.

Overall, the agreement between molecular phylogenetic structure and floral traits highlights the value of integrating ITS and *matK* DNA-barcoding with morphology to address taxonomic complexity in *Rhynchostylis*. Previous molecular studies of *Rhynchostylis* and related *Aerides*-*Vanda* orchids have generally focused either on broad phylogenetic relationships or on variation within individual species, often relying solely on sequence data^12,13,20^. By contrast, the present study examines multiple *Rhynchostylis* taxa and integrates molecular evidence with morphological and phytochemical information. By combining these complementary datasets within a regional context, it provides a more holistic perspective on species boundaries and diversity in the genus rather than proposing a new molecular framework.

###  Antioxidant activity of polyphenolic compounds

Numerous studies have documented strong antioxidant potential in orchids and other medicinal plants, largely associated with polyphenolic compounds. Consistent with these reports, the present study found substantial variation in TPC, flavonoid levels, and DPPH radical scavenging activity among *Rhynchostylis* taxa, indicating marked phytochemical differentiation within the group.

Previous studies have documented substantial variation in phenolic and flavonoid contents among cultivated and wild orchids. For example, Minh et al. (2016) reported that the *Phalaenopsis* hybrid ‘Sunset Beauty’ exhibited the highest TPC, 11.52 ± 0.43 mg GAE g⁻¹ DW) and TFC, 4.98 ± 0.27 mg RE g⁻¹ DW) among six hybrids^69^. In contrast, wild *Rhynchostylis rossii* roots have been reported to contain substantially higher levels of phenolic and flavonoid compounds, indicating strong antioxidant potential^70^. In the present study, *R. gigantea* ‘Chang Phlai’ exhibited the highest phenolic (11.38 ± 0.01 mg GAE g⁻¹ DW) and flavonoid (18.14 ± 0.33 mg RE g⁻¹ DW) contents among the examined taxa. The elevated flavonoid level in this accession may reflect enhanced biosynthetic capacity or the influence of ecological and horticultural factors on secondary metabolite production^71^. Collectively, these findings suggest that cultivated genotypes with hybrid backgrounds may accumulate secondary metabolites differently from wild taxa, potentially reflecting both inherited genetic factors and cultivation conditions.

Comparable patterns have been observed in other orchid species. Natta et al. (2022) documented high phenolic and flavonoid levels in *Dendrobium densiflorum*^71^, while Chand et al. (2016) reported elevated TPC and TFC in leaves of wild *R. retusa* from Nepal^30^. In contrast, the root samples of *R. retusa* analyzed here showed moderate phenolic and flavonoid levels but retained measurable antioxidant activity. Such differences likely reflect variation in plant part, genotype, and growing conditions, which are known to influence phytochemical composition^29^. Notably, *R. gigantea* ‘Chang Phlai’ exhibited the highest DPPH scavenging activity, indicating comparatively strong antioxidant capacity among the taxa examined and, in some cases, exceeding values reported for other orchid genera.

Viewed comparatively, the phytochemical data show patterns broadly consistent with the molecular phylogeny. Lineages resolved as distinct in the ITS and *matK* trees, such as *R. retusa* and *R. coelestis*, also differ in their antioxidant and phenolic profiles, whereas cultivated forms within the *R. gigantea* complex display more similar biochemical values, in line with their close genetic relationships and shared breeding history. Although this comparison is based on observed trait patterns rather than formal clustering analysis, the agreement between phytochemical variation and molecular structure supports the biological relevance of the barcode-based phylogeny.

The integration of molecular phylogenetic analyses, floral morphology, and phytochemical profiling provides a coherent framework for interpreting diversity within *Rhynchostylis*. The evidence consistently distinguishes *R. retusa* and *R. coelestis* as well-defined species outside the *R. gigantea* complex, whereas cultivated forms of *R. gigantea* cluster closely, reflecting shared genetic backgrounds and horticultural selection. Within this complex, accessions differ mainly in quantitative traits rather than deep phylogenetic divergence. Notably, *R. gigantea* ‘Chang Phlai’ emerges as a particularly distinctive accession, combining close phylogenetic affinity with elevated phenolic and flavonoid contents and strong antioxidant capacity. Together, these findings demonstrate that integrative approaches incorporating DNA barcoding, morphology, and phytochemistry are effective for resolving taxonomic uncertainty and for identifying biologically and pharmacologically valuable genotypes within economically important orchid groups. These results offer a useful reference for ongoing floristic studies in Thailand, including the Flora of Thailand Orchidaceae Project, by providing molecularly supported identifications and documented variation within *Rhynchostylis* taxa.

In Conclusion, this study shows that combining ITS and *matK* DNA barcodes with floral morphology and phytochemical evidence provides an integrative framework that helps clarify species relationships within *Rhynchostylis*. The analyses support the recognition of *R. retusa* and *R. coelestis* as distinct taxa, while indicating that diversity within the *R. gigantea* complex is shaped largely by horticultural selection and phenotypic variation rather than deep evolutionary divergence. The consistent identification of *R. gigantea* ‘Chang Phlai’ as a chemically enriched accession further illustrates how integrative approaches can reveal biologically meaningful differences among closely related forms. More broadly, these findings support improved species identification, informed conservation planning, and the sustainable utilization of these economically important orchids within the Orchidaceae.

## Supplementary Information

Below is the link to the electronic supplementary material.

Supplementary Material 1

## Data Availability

The datasets generated and analyzed during the current study are available in the National Center for Biotechnology Information (NCBI) GenBank repository (https://www.ncbi.nlm.nih.gov/genbank/), with direct hyperlinks provided for each accession number listed in Table 3.
