# Supplementary material for: Phylogenetic relationship based on DNA barcodes and comparative analysis of phytochemical contents among Rhynchostylis orchids in Thailand
Source: Sci Rep. 2026 Mar 18;16:13992. doi: 10.1038/s41598-026-44785-x (PMC13133143; doi:10.1038/s41598-026-44785-x)
Supplement: Supplementary file 1 — Supplementary Material 1 [file 41598_2026_44785_MOESM1_ESM.docx]

**
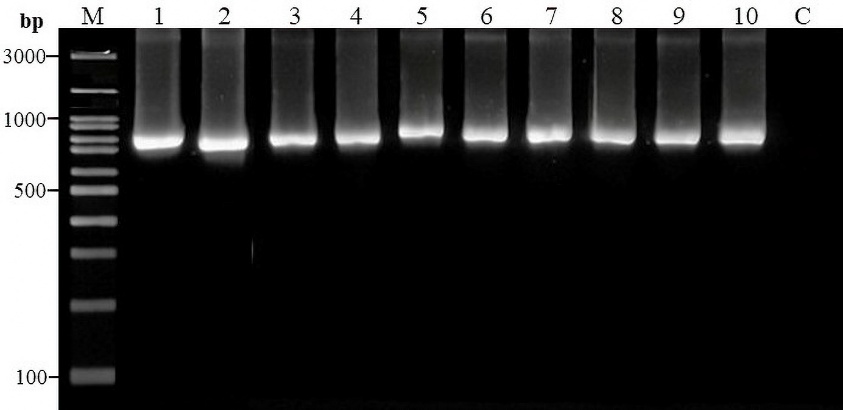
**

**A**

**
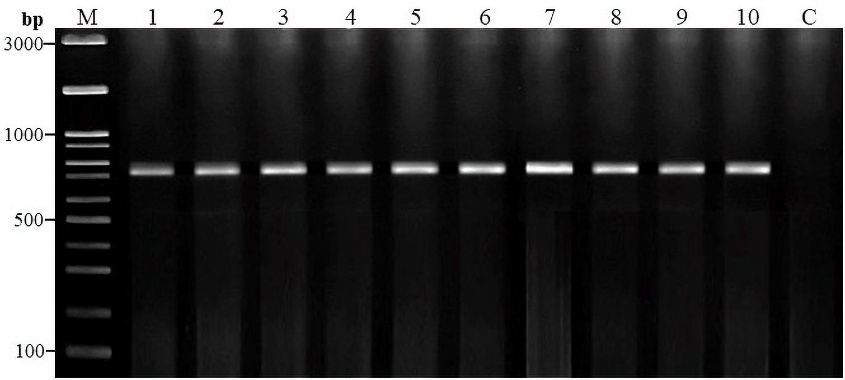
**

**B**

**Supplementary Figure S1.** PCR amplification profiles of the ITS region (A) and the matK gene (B) obtained from the sampled orchid accessions (Replication 1). Lane M: 100 bp DNA ladder; lanes 1-10: R. gigantea, R. gigantea var. harrisonianum, R. gigantea ‘Chang Phlai’, R. gigantea var. rubrum, R. gigantea var. vivaphandhul, R. gigantea ‘Ruxaporn’, R. gigantea ‘Kultana’, R. retusa, R. coelestis, and A. houlettiana; C: negative control.

**
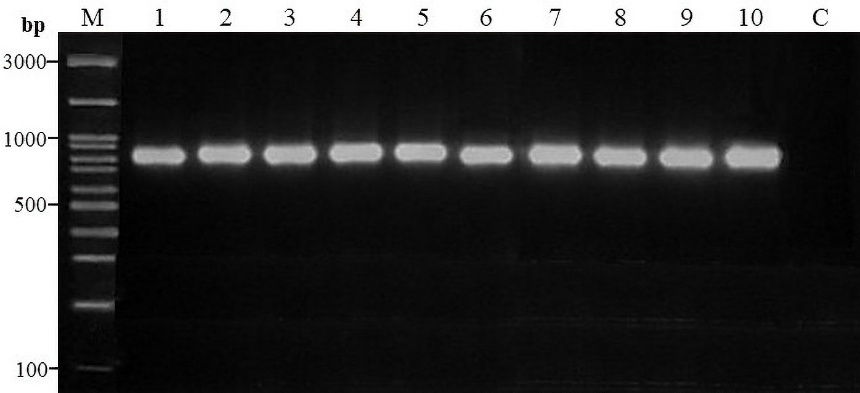
**

**A**

**
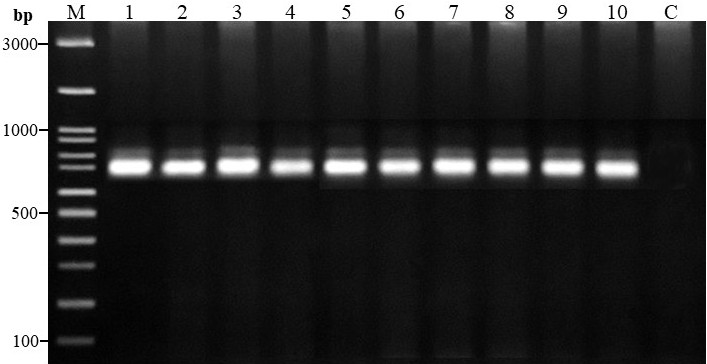
**

**B**

**Supplementary Figure S2.** PCR amplification profiles of the ITS region (A) and the matK gene (B) obtained from the sampled orchid accessions (Replication 2). Lane M: 100 bp DNA ladder; lanes 1-10: R. gigantea, R. gigantea var. harrisonianum, R. gigantea ‘Chang Phlai’, R. gigantea var. rubrum, R. gigantea var. vivaphandhul, R. gigantea ‘Ruxaporn’, R. gigantea ‘Kultana’, R. retusa, R. coelestis, and A. houlettiana; C: negative control.
